# Leveraging the Power of Nondisruptive Technologies to Optimize Mental Health Treatment: Case Study

**DOI:** 10.2196/20646

**Published:** 2020-11-26

**Authors:** Shiri Sadeh-Sharvit, Steven D Hollon

**Affiliations:** 1 Eleos Health Cambridge, MD United States; 2 Center for m2Health Palo Alto University Palo Alto, CA United States; 3 Vanderbilt University Nashville, TN United States

**Keywords:** anxiety, behavioral health, depression, digital health, Eleos Health, mental health, natural language processing

## Abstract

Regular assessment of the effectiveness of behavioral interventions is a potent tool for improving their relevance to patients. However, poor provider and patient adherence characterize most measurement-based care tools. Therefore, a new approach for measuring intervention effects and communicating them to providers in a seamless manner is warranted. This paper provides a brief overview of the available research evidence on novel ways to measure the effects of behavioral treatments, integrating both objective and subjective data. We highlight the importance of analyzing therapeutic conversations through natural language processing. We then suggest a conceptual framework for capitalizing on data captured through directly collected and nondisruptive methodologies to describe the client’s characteristics and needs and inform clinical decision-making. We then apply this context in exploring a new tool to integrate the content of therapeutic conversations and patients’ self-reports. We present a case study of how both subjective and objective measures of treatment effects were implemented in cognitive-behavioral treatment for depression and anxiety and then utilized in treatment planning, delivery, and termination. In this tool, called Eleos, the patient completes standardized measures of depression and anxiety. The content of the treatment sessions was evaluated using nondisruptive, independent measures of conversation content, fidelity to the treatment model, and the back-and-forth of client-therapist dialogue. Innovative applications of advances in digital health are needed to disseminate empirically supported interventions and measure them in a noncumbersome way. Eleos appears to be a feasible, sustainable, and effective way to assess behavioral health care.

## Introduction

### Background

Psychotherapy is on the edge of a transformation driven by technology. More than 50% of adults in high- and middle-income countries will experience a mental disorder in their lifetimes [1]. However, while psychological treatments for most mental disorders have demonstrated efficacy, the quality and effectiveness of mental health care delivery remain inadequate due to multiple reasons, including access, cost, paucity of clinicians trained in empirically-supported models, and the absence of objective and systematic methods for assessing treatments during their delivery [2,3]. Further, health insurance models often limit the number of sessions patients can receive, thereby requiring sustainable outcomes within a relatively short period of time. Treatment delivery models need to be improved to bridge this gap.

One avenue to improving the efficacy and cost-effectiveness of mental health care and increasing patient satisfaction and engagement is integrating technology into clinical practice through patient evaluations and monitoring of the treatment process [4]. Recent digital developments can provide clinicians with nuanced, real-time information to assist their decision-making capacity. Cutting edge technologies can be incorporated into clinical practice for this purpose. Data can be presented to the provider prior to each session to inform them of the patient’s progress, summarize key points from the last session, and prepare them for the upcoming meeting [5]. These data can also inform supervisors, program directors, and other stakeholders to guide clinical decisions, resource allocation, and training.

The goal of this paper is to examine and provide an update on state-of-the-art techniques and methodologies to assess behavioral interventions. We also provide a conceptual framework for collecting and integrating client and treatment data, summarizing, analyzing, and visualizing information to accurately capture the client’s progress and needs. We then demonstrate the utility of a platform incorporating such instruments in the treatment for anxiety and depression.

### Standardized Assessments of Treatment Effects

To formulate a case and assess the impacts of a prescribed intervention, one must rely on data [6]. Technology can be successfully leveraged to provide measurement-based care (MBC), defined as the practice of grounding clinical care in patient data collected throughout treatment [7]. MBC is superior to usual care because it offers several benefits that optimize treatments, such as providing insight into treatment progress, early detection of symptom relapse, and improving outcomes [8]. Evidence indicates that patients whose providers use MBC achieve greater and faster treatment response and symptomatic remission [9]. For instance, 2 validated symptom assessment measures, The Patient Health Questionnaire-9 (PHQ-9 [10]) and the Generalized Anxiety Disorder-7 (GAD-7 [11]), are widely used in practice and have been utilized in thousands of studies and clinical settings globally. As their names suggest, they are very brief, totaling 16 items. They provide an estimation of the patient's depression and anxiety levels and whether these are within the clinically severe, moderate, or normal ranges. Such self-report tools can help clarify the patient’s experience and progress, and provide a real-time signal as to whether the treatment helps them get where they want to be [12].

Despite being the simplest, most cost-effective way of assessing patients’ clinical status, self-report measures in clinical practice have several caveats. Patient compliance with self-assessment is limited, with frequent complaints that questionnaires are cumbersome to complete and repetitive [13,14]. Patients may exaggerate or minimize reports to continue receiving services or avoiding potential consequences of their condition. Therefore, a combination of self-report instruments, clinician ratings, and data from additional resources could likely depict the client’s progress more accurately [15]. In order to make treatments more effective, precise, and relevant to the individuals seeking them, additional measures need to complement surveys and questionnaires. Mental health care must develop innovative technologies that could transform behavioral treatment without disrupting their delivery (eg, consuming time or interrupting the conversation flow) [16,17].

### Machine Learning and Human-Centered Designs in Behavioral Medicine

Timely data are crucial for planning treatment and assessing its effects. Is the client occasionally preoccupied with troubling ideas or does she meet criteria for an obsessive-compulsive disorder? Has the veteran in treatment for insomnia been able to sleep better following the intervention? Does the teen in adolescent-focused therapy feel connected and valued enough in treatment to share their suicidal ideation with their therapist? Clinicians use various methods to collect these data and gauge their predictive value. However, new models should integrate numerous data sources to provide health care that is person-centered, efficient, and targeted to meet patients’ unique needs [18]. Technological developments can ensure information collecting is contextualized, optimized, and translated into clinical insights and actionable decisions by providers and stakeholders [19].

Machine learning (ML) provides unparalleled precision and accuracy in predicting treatment outcomes based on data collected early in treatment, and determining the most successful targets for interventions [20]. ML algorithms can integrate many sources of information to predict the client’s functioning in treatment, such as text used in the therapeutic conversation, the proportion of time each participant talked, conversation turn-taking, and the client’s self-report measures over time [21]. ML can also be used to assess treatment fidelity and the therapeutic relationship, which until recently relied on exhaustive manual work, mostly coded in research trials but not applied to community settings [22]. These capabilities enable timelier identification of trends in patients’ symptoms and the issues troubling them, as well as changes in the therapeutic alliance and the therapeutic relationship [23]. Greater awareness of nuanced changes over the course of treatment, both in the content expressed during the sessions and changes in symptoms throughout the treatment period, has the potential to better inform and better prepare the therapist to provide effective interventions, seek out consultation and support, or complement the current intervention with additional treatment modules as needed.

Increased adherence to evidence-based care predicts improved treatment outcomes [24]. However, ongoing evaluation of treatment progress remains a challenge [25]. Nondisruptive measures were introduced in behavioral medicine over half a century ago. There is documentation as early as the 1970s of audio recordings or videotapes of treatment sessions being routinely used in training, supervision, practice, and consultation [26]. Recordings are widely used in behavioral medicine [27] as video or audio recordings have been mandated in many clinical training programs and routine care [28,29]. Most patients report positive attitudes toward the use of recordings in their treatment [30]. For example, 71% of patients in a recent study were open to considering audio or video recordings of their treatment sessions. The patients’ comfort with recording was not associated with treatment refusal, duration, or outcomes [31].

Sophisticated algorithms for voice analysis through natural language processing (NLP) allow for the detection of trends in the conversation’s sentiment, content, and synchrony of participants. Insights from session recordings can inspire behavior change, informing the clinician of metrics relevant for both the process and the content of the intervention. Of note, data collected passively during the regular course of treatment in a nondisruptive manner can inform with respect to therapist variables as well. These data provide a broader, more nuanced consideration of how patient, treatment, and clinician variables interact to achieve treatment effects [32]. As such, methods that passively assess and integrate multiple variables at the same time can significantly affect treatment engagement, adherence, and outcomes. When ML models integrate several data resources and are scientifically robust, they can be implemented in a platform offering artificial intelligence (AI) information and prediction [33].

A plethora of empirical evidence suggests that within-session process variables predict the patient-therapist connection, the patient’s mood and anxiety, and the content of the patient’s interests, strengths, concerns, and dilemmas [34,35]. As such, data derived from session recordings can serve as objective markers of the treatment process and inform the clinician where to head next [36]. The patient/therapist listening ratio, number of cross-talks, and silences all reflect the nature of the clinical relationship, therapeutic alliance, and the patient’s engagement with the treatment. Evidence shows that treatments in which patients speak about themselves and are engaged in the conversation are particularly likely to maintain momentum [35]. Patient involvement in the session and therapist active listening can be observed by conversational interaction when the patient and the therapist take turns speaking, when there are few extended pauses, and when neither party overrides the other. Silences are effective when they are used in later sessions for brief periods. Long silences by the patient reflect a lesser sense of connection, affecting attrition, adherence, and outcomes [34].

Within-session content variables, such as data on most commonly used themes and affective tone, can inform the therapist, stakeholders, and policymakers in addressing underlying perpetuating factors. Integrating sentiment and the themes discussed can further inform stakeholders regarding treatment progress [37]. Patients that express less emotional content tend to rate the therapy as less helpful and their connection with their therapist as weaker [35]. Further, greater therapist insight into the interests, concerns, and experiences of the patient predicts whether they will reach the outcomes desired by each party [38,39].

### Current Implementation of Digital Tools to Augment Behavioral Medicine Outcomes

Although their potential role in optimizing treatment delivery has been proposed, in-session and between-session data are not collected regularly, nor have they been integrated into mental health care services as of yet. Barriers often cited are limited clinician time to administer, collect, and analyze data, and concerns that the administration of measures would interfere with rapport and the therapeutic alliance [40]. For a digital tool to be maximally effective, it needs to collect information passively, without increasing therapist burden or reducing face-to-face communication [41]. Additionally, these data should be provided to therapists via a platform that is straightforward and easy to use, with clear visualization and comparison to earlier sessions. Such information presented in a timely fashion can inform decisions regarding resource allocation, such as increasing treatment dose, revisiting the level of care, and team consultation [7]. Further, aggregated data on treatment progress and outcomes can be used by providers and clinic directors for quality assessment.

## The Eleos Health Platform

### Description

Eleos Health is a therapy intelligence engine designed to provide intervention insights and inform clinical decision-making. The platform collects key metrics from treatment sessions and integrates them with standardized assessment scales, leveraging insights developed through ML and NLP analysis of large treatment datasets. The Eleos platform integrates subjective and objective measures of the treatment process, the patient-therapist communication, and outcomes into AI software. In the following case illustration, we demonstrate how objective measures of treatment process and content derived from session recordings can be integrated with patient self-assessments in real time to shape clinical insights and decisions in a positive direction. Eleos Health is a digital platform designed to integrate multiple patient data points to present providers with a comprehensive picture of the client’s progress in treatment. The platform is used in an app that can be used on a mobile device or desktop computer for in-person meetings or embedded within teleconferencing programs. The platform applies voice analysis to describe and summarize events throughout the treatment meeting, including the language used by the therapist and the patient. These data are complemented with weekly outcome monitoring through self-report assessments.

### Case Example

The following case example illustrates the pilot use of an AI platform hosted by Eleos Health to collect and analyze the content of treatment sessions. The patient described signed consent for using de-identified treatment data prior to beginning the treatment and gave permission for the following text. All identifying information has been changed.

Kyle was a 24-year-old Latino man. He was born and raised in a small suburb next to a big metropolis. The youngest of 4 children, he described a very warm and strongly connected family growing up. He had graduated from a liberal arts college the previous year and found a job in a small startup company. He moved to a new city, where he lived with 3 roommates. Kyle described that in the past 18 months, since his junior year in college, he felt concerned about his future and unsure about which career path to choose. His friends and family recommended that he seek counseling, but after calling several therapists who did not have a slot, he did not begin treatment. Instead, he focused on his eating and physical activity, and thought that his new routine supported his transition into his new job. Kyle recently decided to seek treatment again after receiving a promotion at work. His boss moved out of town and the CEO of the company offered Kyle her position. Kyle accepted the promotion but became very anxious and had a hard time concentrating at work. He also reported sleep problems and that the healthy lifestyle he had worked hard to develop had been derailed.

When Kyle reached out to Dr. Davis, the therapist suggested incorporating a platform in his treatment that could record and analyze their treatment sessions. The therapist also suggested that the system send Kyle weekly assessments to enhance the therapist’s understanding of how best to help him. Kyle was skeptical that a digital platform could inform his therapist beyond the treatment session per se, but decided he had little to lose by trying it out. He signed a consent form that the therapist had sent him, which included an authorization to use a HIPAA-compliant platform named Eleos. Treatment was conducted in a blended fashion, integrating in-person and remotely delivered meetings. The Eleos platform provides voice analysis of the sessions, regardless of their delivery method.

Kyle received a text message from the system before the first session, requesting that he complete the PHQ-9 and the GAD-7. He completed the 16 items on his phone within a few minutes, which served as his baseline scores. When he sat in the therapist’s office in the first session, she pointed at a mobile device that she would use to record and analyze their meeting. Kyle’s therapist was very welcoming and helped him solidify his goals, which were to feel less anxious at work and happier after work hours. The therapist mentioned that Kyle’s PHQ-9 score was 18 and that his GAD-7 score was 15, indicating that his depression and anxiety were both in the severe range [10,11]. Since Kyle’s insurance covered only 12 sessions, the therapist explained that she would provide cognitive behavioral therapy, focusing on his interpretations of ongoing events and how these beliefs affected his emotions and behaviors. Given the severity of his symptoms, she also referred him for medication evaluation.

**Figure 1:**
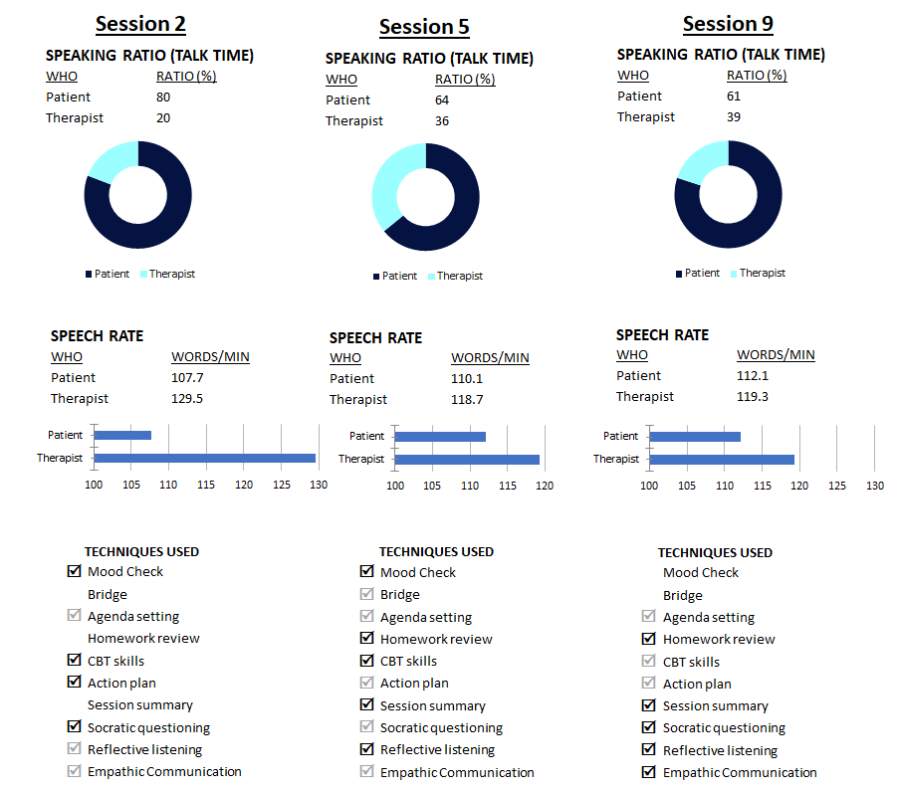
Screenshots illustrating some of the Eleos Health platform process features. CBT: cognitive behavioral therapy; Speaking Ratio: proportion (%) of time spent speaking during the session; Speech Rate: speed of words per minute; Techniques Used: intervention strategies employed by the therapist during the session and automatically identified by the platform. Techniques used 3 times or more are indicated with a black checkmark, techniques used once or twice are denoted with a grey checkmark, and interventions not employed in the session do not have a checkmark.

After their second session, Dr. Davis reviewed the reports she received from Eleos, the therapeutic intelligence platform she had been using. She was surprised to see that in their last session, she spoke in only 20% of the session and that she was speaking much slower than Kyle (Figure 1). The speech rate difference indicated to Dr. Davis that Kyle was speaking hurriedly and reflected her attempt to “slow him down” to reflect on his maladaptive assumptions and ingrained interactional patterns. However, she was wondering whether she could share these observations with Kyle more explicitly. These data helped the therapist understand that Kyle was experiencing significant mood, anxiety, and stress symptoms, but that in order to help Kyle achieve his goals, their synchrony during the session would have to be substantially improved [35]. A review of the process metrics provided insight into the issues troubling Kyle and the inaccurate beliefs and maladaptive information processing strategies that precipitated and maintained his cognitions [42]. A review of his most frequently used words indicated that he tended to use verbs, adjectives, and expressions associated with negative self-esteem, such as “failed,” “not as good as,” “disappointed,” and “messed it up,” when he talked about his work. However, when he talked about his relationships, he tended to use anxiety words, such as “stressed,” “pressured,” “overwhelmed,” and “toxic.” The system flagged these words and phrases, as they approximate depressive and anxiety symptoms and may reflect Kyle’s subjective experience, thereby enriching the self-report data collected between sessions. In addition, 3 of Kyle’s most used phrases were “shoulds” (eg, “have to,” “I must,” “should have known better”), which the system automatically analyzed and flagged. The therapist realized that Kyle was experiencing 2 distinct phenomena: At work, he felt like an imposter and worried about his functioning, whereas outside of work, he was distressed by blurred boundaries in his interpersonal relationships. Dr. Davis also realized that she was using little Socratic questioning, which may have reduced Kyle’s ability to re-examine his assumptions and information-processing skills [43]. However, she did observe her use of reflective listening methods often, mirroring and reframing what Kyle had said, which she was content with [44,45]. Dr. Davis brought the case to her weekly group consultation meeting and received feedback and advice from her peers that she intended to implement in the next few sessions.

**Figure 2:**
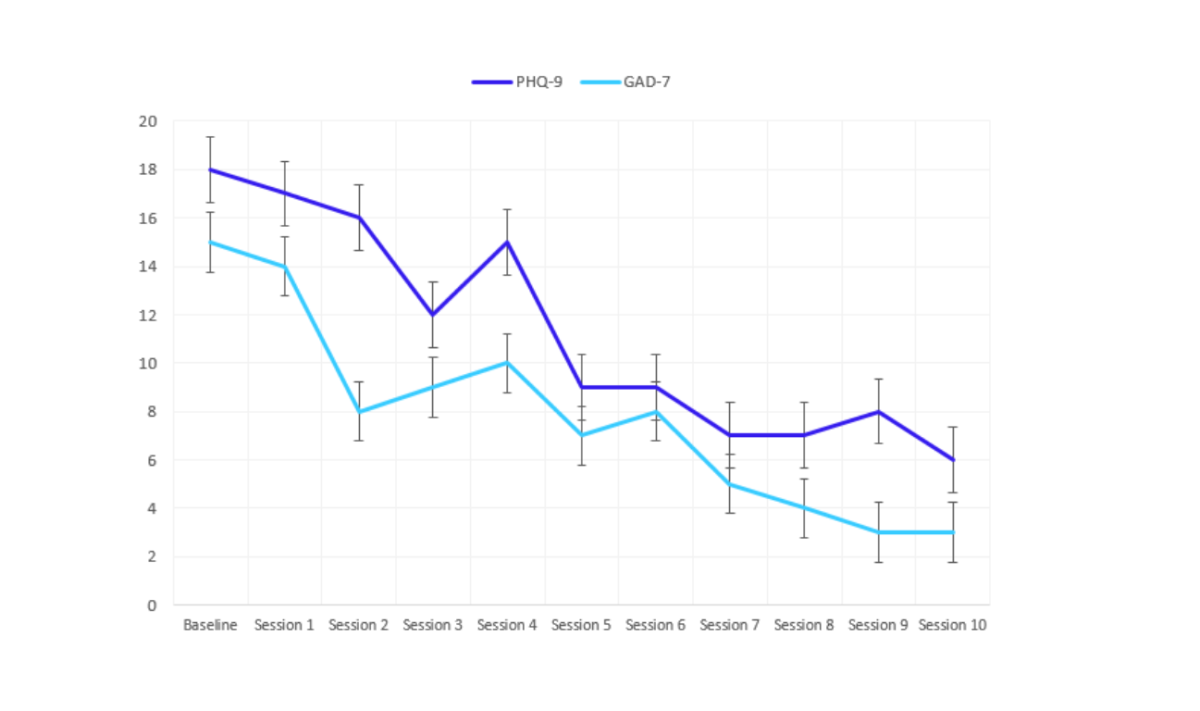
Patient self-monitoring data graphed on the Eleos platform. GAD-7: Generalized Anxiety Disorder-7; PHQ-9: Patient Health Questionnaire-9.

In the following meeting, Dr. Davis shared with Kyle the insights she had gleaned from the platform. She described the 2 clinical issues—his depression regarding work and his anxiety regarding relationships—and asked him to prioritize his goals. Dr. Davis’s presentation of his challenges helped Kyle reflect on them, and he decided to first focus on his self-esteem at work. He learned how to observe, identify, and challenge negative perceptions of himself, and reported very quick improvements in his mood. Next, he was able to undertake the same process regarding several relationships that he felt had not been reciprocal and gratifying. He was pleased with his progress in treatment and felt happier and more relaxed. Kyle also liked that he could complete short assessments on his phone and got into the habit of doing this on his commute home from work when he had a few minutes to spare. Similarly, Dr. Davis appreciated the symptom-tracking feature, which let both her and her patient easily see how he was doing symptom-wise (Figure 2). In the session analytics reports, Dr. Davis also observed an improvement in the therapeutic alliance: She was able to incorporate more open-ended questions, Kyle was more receptive of her questions and comments, and their speech rate was more in sync with one another (Figure 1).

After 10 sessions, Kyle was not only feeling less depressed and anxious but was also receiving praise from his coworkers and his boss. He was able to strengthen his friendships and practice new skills for boundary-setting and interpersonal effectiveness. As treatment termination neared, the last sessions focused on brainstorming strategies for maintaining his progress, particularly since the workload at his company continued to increase.

## Conclusions

Psychological interventions for mental disorders were found effective in numerous research trials. However, gaps in training, availability, access, dissemination, and cost impede the successful delivery of these interventions [46]. These are enhanced by privacy and data storage regulations, which are key for maintaining clients’ rights and trust [47]. The ever-growing demand for mental health care requires optimizing clinician decision-making using data collected passively [48]. Our goal was to highlight key issues for optimizing available mental health services and to demonstrate how a therapeutic intelligence platform can support this process.

This paper presents a novel approach to collecting comprehensive data on treatment progress. The implementation of ML models and AI in behavioral health care is a rapidly moving and innovative field, with the potential to significantly improve screening and clinical outcomes [49,50]. Accurate data can provide more information about the patient and can be translated into clinical decisions faster. Experts strive to base their decisions on data. Primary physicians, heart surgeons, physiotherapists, and other health professionals all function better when they review the patient's most recent tests and laboratory results prior to the appointment. Therefore, using real-time data is equally important in enhancing the work of mental health professionals [51]. Technologies that are scalable, cost-effective, and that enhance quality without burden can help therapists harness their efforts into providing more effective interventions with increased fidelity to data-informed treatments. Setting out clear performance targets in the training, provision, and implementation of evidence-enhanced treatments will enable health care services to continually improve. Nondisruptive measures are poised to ensure nearly effortless data collection, and innovative methods that inform clinicians and other stakeholders of the patient’s progress will likely make treatments more relevant and engaging [52].

Some caveats to the model described here should be mentioned. The Eleos platform was illustrated through its use in an outpatient setting, with a client who was relatively high functioning and a therapist that was tech-savvy and interested in using novel digital programs. Usability testing, user reviews, and long-term engagement with any product are key to realizing the practicality and helpfulness of new tools over time [53]. Further, evidence from more case reports, randomized controlled trials, and meta-analyses is needed to render these technologies pertinent, empirically supported, and easily applied in clinical settings.

The effective implementation of mental health care requires new approaches for developing, implementing, and evaluating interventions. A person-centered approach that capitalizes on greater data insights will certainly enhance the therapeutic process. Technology can help make efforts in this direction scalable and more efficient, thereby increasing the effects of behavioral interventions and reducing the burden of mental health problems worldwide.

## Abbreviations

AI: artificial intelligence
GAD-7: Generalized Anxiety Disorder-7
MBC: measurement-based care
ML: machine learning
NLP: natural language processing
PHQ-9: Patient Health Questionnaire-9
